# Challenges and Barriers to Medical Research Among Medical Students in Saudi Arabia

**DOI:** 10.7759/cureus.59505

**Published:** 2024-05-02

**Authors:** Khalid M Alduraibi, Mohammed Aldosari, Abdulrahman D Alharbi, Abdulaziz I Alkhudairy, Mohsen N Almutairi, Nawal S Alanazi, Mohammad S Almosa

**Affiliations:** 1 College of Medicine, King Saud bin Abdulaziz University for Health Sciences, Riyadh, SAU; 2 Family Medicine, King Abdulaziz Medical City, Riyadh, SAU; 3 Family and Community Medicine, King Saud University, Riyadh, SAU; 4 College of Medicine, Northern Border University, Arar, SAU

**Keywords:** barrieries to medical research, saudi arabia, challenges, medical research, medical students

## Abstract

Background: The pivotal role of research in medicine is undeniable, as it is vital for the progress of healthcare methods and patients' well-being. This notwithstanding, medical and dental students in Saudi Arabia face many barriers that prevent them from participating in research activities. This research aims to reveal the impediments that are particularly relevant, with select challenges and barriers being mentioned, such as time issues, the inadequate supply of resources, and insufficient training and guidance.

Materials and methods: By using a cross-sectional study, the researchers have provided a questionnaire for medical students across multiple Saudi Arabian medical colleges via the online platform. The IBM SPSS Statistics for Windows, Version 23 (Released 2015; IBM Corp., Armonk, New York, United States) was used for data analysis, which leaned clearly on the descriptive statistical part, using a chi-square test to investigate the association between two categorical variables.

Results: There were 469 total participants, and data analysis clearly showed that lack of statistical skills (74.2%), time constraints (73.3%), and research topic selection (71.4%) were the most major obstacles to research participation. Even though the same barriers existed, a significant percentage of students (75.5%) definitely showed interest in the research, with 89.6% of the students recognizing the importance of research in the medical field. Furthermore, it should be highlighted that the female students showed a stronger positive attitude toward research than the male students (70% vs. 58.3%).

Conclusion: The results highlight the necessity for the improvement of the medical education curriculum within Saudi Arabia, including the creation of a research participation system for the students. Through learning strategies emphasizing the importance of research, mentorship programs, and providing resources to the students, there will be an increase in their participation and success in the research. This will lead to an enriching medical research environment.

## Introduction

Research is important to improve medical knowledge, gain new knowledge, decrease misdiagnoses, come up with new management of an illness, and use preventive medicine [1,2]. Research starts with an idea that has questions that need to be answered, a literature review, finding the objectives of the study, choosing materials and methods (study area/settings, study subjects, design, calculating sample size, and acquiring sampling technique), selecting tools that are valid and reliable to achieve the goals of the study, and finally writing the rest of the proposal, obtaining approval of the proposal, data collection, and analysis [1]. It's essential for medical and dental students to learn and write medical papers because they play a vital role in the development of future healthcare [3]. Another research found that only one in five undergraduate medical students do not have enough time to conduct research during their medical years. It also has been found that medical students suffer from inadequate training and supervision [4].

In a study by Arshad et al. (2021) it was found that out of 215, 128 (59.5%) had a previous research experience mostly as a part of the curriculum and reported lack of resources and lack of interest as the main barriers [5]. Also, a study by Hart et al. (2022) found that the major barrier to successful research projects was the lack of time allocated for research activities. Furthermore, they suggest that the experience of the research supervisor had a significant impact. For example, novice supervisors reported higher rates of unexpected project delays and data acquisition problems compared with more experienced ones [6].

Among global studies, the challenges and barriers vary depending on the country. In a study by Chen et al. (2023) in China, the most common barrier among medical students was a lack of proper mentoring opportunities and knowledge [7], while a study in Nigeria found that the commonest barriers to research were financial and time availability [8]. Last, a study by Assar et al. (2022) done in six different Arab countries found a lack of access to lab equipment for lab research (68.1%), the priority of education over research (66.8%), and a lack of time because of educational priorities (66.1%) were among the most perceived barriers towards research practice [9].

This study aims to identify the obstacles that medical and dental students face when conducting medical research. Furthermore, the study aims to explore the reasons why medical students may not participate in research activities, despite the importance of research in increasing medical knowledge. The study also aims to identify challenges that students face when conducting research, such as lack of time, resources, and mentorship. By identifying these challenges and barriers, the study aims to provide recommendations for improving research opportunities and support for medical students.

## Materials and methods

Study area/setting

A cross-sectional study was conducted at King Saud bin Abdulaziz University for Health Sciences, Riyadh, Saudi Arabia. A self-administered online-based questionnaire was utilized at various colleges of medicine in Saudi Arabia. Medical students were selected through a non-probability convenient sampling technique. The questionnaire comprised a section recording demographics, barriers, and attitudes toward medical research.

Inclusion and exclusion criteria

The inclusion criteria encompass medical students in Saudi Arabia, both female and male. Excluded from the study are students from other specialties such as nursing, applied medical sciences, premedical, and basic years medical students.

Sample size

The medical students’ population is estimated to be 20342, so the estimated sample size is 469 with a confidence level of 95% and a 5% margin of error.

Sampling technique

A non-probability convenient sampling technique was used by including all those who meet the inclusion criteria.

Data collection methods, instruments used, and measurements

A cross-sectional study was conducted in many colleges of medicine in Riyadh, Saudi Arabia, to investigate the barriers and challenges toward medical research among medical students; a self-administered online-based questionnaire was used. Male and female medical students were selected through a non-probability convenient sampling technique.

Data management and analysis plan

The sections of the questionnaire were taken from another study that was done at King Khalid University in Abha, Saudi Arabia [2]. The first section of the questionnaire consists of demographics such as age, gender, medical year, and university.

The second part is about barriers experienced by medical students in conducting research and involves different questions such as lack of time, inadequate skills in doing research, inadequate skills in statistics methods, lack of finance, limited database access, lack of interest, difficulty in choosing the topic, getting permission from the ethical committee, difficulty in writing a proposal, difficulty in analysis, difficulty in writing a report, lack of rewarding and/or motivation, and did you find any motivation from faculty in your college to conduct research?

The third part is about interest in doing research, continuing to work in research after graduation, the importance of medical research, participating in any research, awards for your research, and any courses that were done in research.

Data analysis

The collected data was analyzed using IBM SPSS Statistics for Windows, Version 23 (Released 2015; IBM Corp., Armonk, New York, United States).

This study received ethical approval from the Institutional Review Board of King Abdullah International Medical Research Center, Ministry of National Guard Health Affairs, Riyadh, Kingdom of Saudi Arabia (IRB/1361/23).

## Results

A total of 469 medical students were included in this study. The median age of theirs was 23 years (IQR: 21-23), and most of them were in the age group of 21 to 23 years: 268 (57.1%), 115 (24.5%) were in the age group of 24 to 26 years, and 86 (18.3%) were in the age group of 18 to 20 years. When we assessed the academic level of participants, we found that about 144 (30.7%) of the study population were fifth-year medical students, 91 (19.4%) were sixth-year medical students, 90 (19.2%) were in the fourth year, 76 (16.2%) were in the third year, and only 68 (14.5%) were at the second year. The grade percentage average (GPA) for most of the students was 4.50-4.74 and more than 4.75 (115 (24.5%) and 111 (23.7%), respectively) (Table 1).

**Table 1:** Socio-demographic characteristics of the study participants (n = 469) N: Frequency; %: Percentage; GPA: Grade percentage average

Variable	Categories	N (%)
Gender	Male	206 (43.9)
	Female	263 (56.1)
Age (in years)	18-20	86 (18.3)
	21-23	268 (57.1)
	24-26	115 (24.5)
Medical year	2nd year	68 (14.5)
	3rd year	76 (16.2)
	4th year	90 (19.2)
	5th year	144 (30.7)
	6th year	91 (19.4)
GPA	Less than 4	85 (18.1)
	4-4.24	66 (14.1)
	4.25-4.49	92 (19.6)
	4.50 4.74	115 (24.5)
	More than 4.75	111 (23.7)

**Figure 1:**
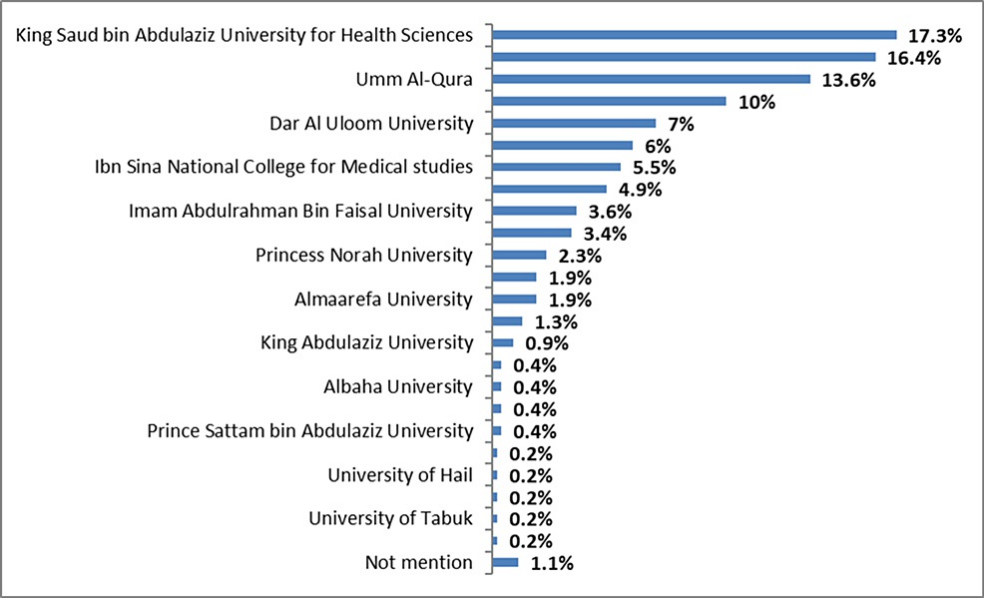
Universities of the study participants %: Percentage

When we asked about the universities that our participants studied, we reported that most of the participants studied at King Saud bin Abdulaziz University for Health Sciences and Northern Border University (81 (17.3%) and 77 (16.4%), respectively), followed by Umm Al-Qura University 64 (13.6%), King Saud University 47 (10%), Dar Al Uloom University 33 (7%) and Al-Imam Muhammed Ibn Saud Islamic University 28 (6%). Other universities are shown in Figure 1.

When evaluating the medical students’ responses to barriers that they faced in conducting research, barriers were examined by the responses to 14 items in Table 2. Based on their answers, inadequate skills in statistics methods was the most reported barrier in 348 (74.2%) followed by lack of time in 344 (73.3%), difficulty in choosing a topic in 335 (71.4%), and difficulty in analysis in 326 (69.5%). Inadequate skills in doing research and limited database access were mentioned by 304 (64.8%) and 299 (63.8%) of the participants, respectively. About 280 (59.7%) of the participants experienced difficulty writing reports and 277 (59.1%) faced a barrier to getting permission from the ethical committee. More than half of the participating students stated that lack of reward and/or motivation and difficulty in collecting data (273 (58.2%) and 272 (58%), respectively) were the barriers they faced when conducting research. The common barriers experienced by medical students in conducting research are shown in Table 2.

**Table 2:** Barriers experienced by medical students in conducting research N: Frequency; %: Percentage

Barriers	Yes	No
	N (%)	
Lack of time	344 (73.3)	125 (26.7)
Inadequate skills in doing research	304 (64.8%)	165 (35.2)
Inadequate skills in statistics methods	348 (74.2)	121 (25.8)
Lack of finance	235 (50.1)	234 (49.9)
Limited database access	299 (63.8)	170 (36.2)
Lack of interest	204 (43.5)	256 (56.5)
Difficulty in choosing a topic	335 (71.4)	134 (28.6)
Getting permission from the ethical committee	277 (59.1)	192 (40.9)
Difficulty in writing proposal	247 (52.7)	222 (47.3)
Difficulty in collecting data	272 (58)	197 (42)
Difficulty in analysis	326 (69.5)	143 (30.5)
Difficulty in writing report	280 (59.7)	189 (40.3)
Lack of reward and/or motivation	273 (58.2)	196 (41.8)
Find any motivation from faculty in your college to conduct research	266 (56.7)	203 (43.3)

Regarding the students' attitude toward medical research; more than two-thirds of them: 354 (75.5%) had an interest in doing research, and a slightly lower percentage of them: 324 (69.1%) planned to continue working with research after graduation. The majority of the participating students 420 (89.6%) believed that medical research is important and 362 (77.2%) had participated in research before. Only 167 (35.6%) of the participants published their research and 128 (27.3%) were awarded for their research. About two-thirds of the participants, 312 (66.5%), had attended a course in research, and 287 (61.2%) had an idea that they think is good for research (Table 3).

**Table 3:** Attitude toward medical research N: Frequency; %: Percentage

Question	Yes	No
	N (%)	
Do have an interest in doing research?	354 (75.5)	115 (24.5)
Do you plan to continue working with research after graduation?	324 (69.1)	145 (30.9)
Do you believe in the importance of medical research?	420 (89.6)	49 (10.4)
Have ever participated in any research?	362 (77.2)	107 (22.8)
Did you publish it?	167 (35.6)	302 (64.4)
Did get awarded for your research?	128 (27.3)	341 (72.7)
Have you ever attended a course in research?	312 (66.5)	157 (33.5)
Do you have an idea that you think it's good for research?	287 (61.2)	182 (38.8)

**Figure 2:**
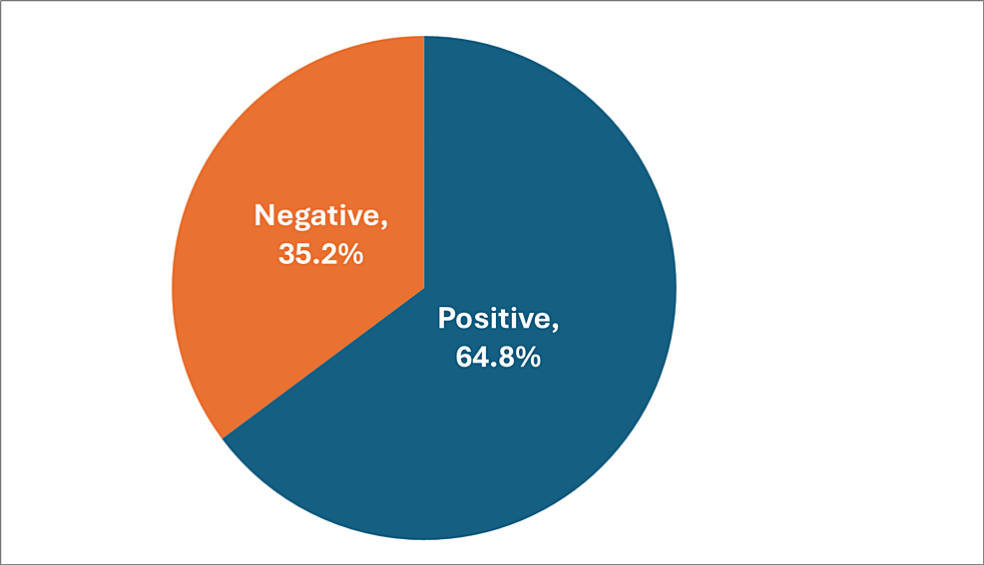
Attitude of participants toward research %: Percentage

The median attitude score for the students was five (IQR: 4-6) out of eight, and nearly two-thirds of the students (n = 304, 64.8%) had a positive attitude toward research (i.e., with a total attitude score > 4), while 35.2% (n = 165) of them had a negative attitude with a total score ≤4 (Figure 2).

Regarding the association between socio-demographic factors and attitude toward medical research; gender was found to be significantly associated with a level of attitude (P = 0.008), with females tending to have a positive attitude more frequently than males (n = 184, 70% vs. n = 120, 58.3%). Age, medical year, and GPA were not found to be significantly associated with attitude toward medical research (P = 0.735, 0.301, and 0.113, respectively) (Table 4).

**Table 4:** Factors associated with attitude toward medical research *P < 0.05, significant N: Frequency; %: Percentage; GPA: Grade percentage average

Variable	Categories	Attitude level: N (%)		P-value
		Positive	Negative	
Gender	Male	120 (58.3)	86 (41.7)	0.008*
	Female	184 (70)	79 (30)	
Age (in years)	18-20	53 (61.6)	33 (38.4)	0.735
	21-23	174 (64.9)	94 (35.1)	
	24-26	77 (67)	38 (33)	
Medical year	2nd year	39 (57.4)	29 (42.6)	0.301
	3rd year	48 (63.2)	28 (36.8)	
	4th year	57 (63.3)	33 (36.7)	
	5th year	103 (71.5)	41 (28.5)	
	6th year	57 (62.6)	34 (37.4)	
GPA	Less than 4	47 (55.3)	38 (44.7)	0.113
	4-4.24	39 (59.1)	27 (40.9)	
	4.25-4.49	59 (64.1)	33 (35.9)	
	4.50-4.74	82 (71.3)	33 (28.7)	
	More than 4.74	77 (69.4)	34 (30.6)	

## Discussion

Undergraduate medical students often lack research knowledge and interest, as shown in several studies. Understanding the research process, which includes data analysis, study design, and results dissemination, may be difficult for many medical students. Furthermore, medical students may not be as engaged or interested, which might lower their incentive to seek research opportunities. Since research is the foundation of medicine and the provision of quality healthcare to the public, this lack of knowledge and motivation may be detrimental to the health sector [10]. The current study of the challenges and barriers that stand in the way of medical research among medical students provides empirical data on how busy or free this particular group is in the research at the given moment.

The study reflects major research barriers reported among medical students, like deficiency of training in statistics, absence of time, and difficulty in specific topic selection and this is revealed in global studies. Moreover, a systematic review and several cross-sectional studies have conferred similar conclusions, reiterating time limitations, insufficient research training, and limited funding to be the main hindrances to medical students' involvement in research activities [11-13]. These findings symbolize a problem for medical education that is cross-border involving the integration of research training and chances in the curriculum. Addressing these obstacles might include the delivery of research methodology courses at the early curriculum level and workshops on statistics and research skills. Furthermore, the development of mentorship programs can help to pick up research topics for students and tackle the research process [14].

The study demonstrated positive attitudes toward research, with students expressing interest in continuing research in the future, reflecting a broader understanding of the benefits of research in medical practice. When compared to these other studies, it is evident that a majority of medical students believe that research improves clinical practice and that research ought to be incorporated into the curriculum of medical schools [15,16]. These findings reveal that most students appreciate research but they may not be able to relate this interest to practical experience. Medical schools can help students bridge the gap between engaging in research activities and the effective dissemination of their findings by offering various platforms for students to showcase their work. These platforms include organizing student research forums or academic conferences where students can present their research projects [17]. Ensuring a good attitude toward undergraduate research and preventing any obstacles that could stop medical students from getting involved in effective medical research actions are crucial [18]. The knowledge of reading scientific publications and clinical decision-making, which is based on critical evaluation of scientific data, should be an integral part of the education of medical students, regardless of their future careers [18]. It has now become crucial to increase research funding, improve salaries, and enhance incentives for productivity for medical students, as well as for those enrolled in post-graduate and dual degree programs. This strategic approach aims to boost motivation and support for those at the forefront of medical research and education [19].

One of the main findings of the study, that there was a positive correlation between gender and a favorable research attitude, is an issue that deserves to be studied further. The results also correspond to other studies on the matter that focus on factors such as gender, academic year, and GPA in explaining how a student's attitude and activity toward research are formed [20]. This study suggests that girls are much more positive towards research in general, and the trend may probably be affected by some factors, including academic atmosphere, mentoring chances, and personal goals. The findings of these studies can be contrasted with an evidence-based study report that showed no association between female and male students with their attitudes and knowledge about medical research [15]. Analogous results have been revealed in another work carried out at Ain Shams University [21]. However, our findings were consistent with a study conducted in Pakistan, which found that gender had no effect on understanding of health research, but men significantly outperformed women in terms of attitude [22]. In contrast, a Saudi study found that female students were more enthusiastic about research than their male counterparts. Nevertheless, the relationship between research attitude and academic performance (e.g., GPA) is multifaceted and demonstrates that while high academic level may correlate positively with research attitude, this categorical factor is not the only one that plays a role in this area [13,16,20].

The challenges and barriers identified necessitate targeted interventions to enhance research participation among medical students. Research training should be a compulsory part of teaching in the medical curriculum, where students will get exposure to research practical work and obtain research funding and mentorship as well, which is paramount to solving the problem. Another way to accomplish a higher research engagement amongst medical students would be embedding a research-based curriculum into the medical school [14,20,23]. This approach not only addresses the immediate barriers to research participation but also lays the groundwork for a future healthcare workforce that is proficient in research and evidence-based practice.

The study presents several notable strengths that contribute significantly to the literature on medical education and research. First of all, the cross-sectional survey design implemented across different colleges of medicine in Saudi Arabia gives a general picture of the challenges and attitudes of medical students toward medical research of a diverse group of students. Moreover, the fact that the study not only considers the range of barriers to research and attitudes toward it but also addresses this gap in the current literature provides valuable information to educators and policymakers who want to allow more students to be involved in research. Nevertheless, the study of the various limitations and disadvantages is indispensable. The convenient sampling method, non-probability, which allows the easy gathering of data, could also introduce selection bias by the choice of participants, which might not represent the exact cross-section of medical students in Saudi Arabia. Moreover, a survey methodology based on a cross-sectional design captures the beliefs and experiences of the students at a particular moment in time, hence confounding the consideration of how those attitudes and experiences could transform over four years of medical education. You need to make sure that this is also one of the factors that limit you from establishing any causal relationship between the barriers and research choices. Finally, the self-reported data employed throughout might be skewed because of the response bias, and students might exaggerate their interest in research or understate the obstacles they may experience by social desirability.

## Conclusions

This study focuses on the barriers and views of medical students towards medical research in Saudi Arabia. The results indicate that there is a lot of interest in research but at the same time, the students are encountering challenges involving inadequate statistical skills, lack of time, and selection of research topics. Similarly, the researchers' zeal in the face of these difficulties demonstrates an understanding of the vital function research plays in medical education and practice. For more effective handling of this phenomenon and avoiding these obstacles, medical schools should engage their students in comprehensive research methodology education, organize courses on time management, set up mentorship programs for students and already experienced researchers, and allow more research opportunities with financial assistance. Through these initiatives not only will students get a chance to work at the research level but also the whole field of medical science will develop eventually, this will in turn positively affect the standard of patient care. Through the use of targeted intervention approaches that are aligned with the identified obstacles and leverage favorable attitudes toward research, medical schools will be able to create a more conducive environment for innovation in research and collaboration.
